# Avoidable Cancer Mortality in Germany Since Reunification: Regional Variation and Sex Differences

**DOI:** 10.3389/fpubh.2019.00187

**Published:** 2019-07-10

**Authors:** Ronny Westerman, Michael Mühlichen

**Affiliations:** ^1^Competence Center Mortality-Follow-Up, German National Cohort (GNC), Federal Institute for Population Research, Wiesbaden, Germany; ^2^Federal Institute for Population Research, Wiesbaden, Germany

**Keywords:** demography, avoidable mortality, cancer mortality, regional differences, gender gap, Germany

## Abstract

**Background:** Regional comparisons of cancer-related mortality in Germany are traditionally focused on disparities between East and West Germany. Recent improvements in all-cause and cancer-related mortality show a diverse regional pattern beyond the known East-West mortality divide. A generalized approach of the avoidable/amenable cancer mortality definition is applied for suitable regional comparisons of long-term trends.

**Methods:** Standardized death rates of preventable and amenable cancer mortality for men and women were computed for the period 1990–2014 to observe sex-specific excess mortality due to specific cancers after the German reunification. For regional comparison, three German super regions were defined in Eastern, Northwestern, and Southwestern Germany to account for similarities in long-term regional premature and cancer-related mortality patterns, socioeconomic characteristics, and age structure.

**Results:** Since preventable and amenable cancer mortality rates typically have driven the recent trends in premature mortality, our findings underline the current regional pattern of preventable cancer mortality for males with disadvantages for Eastern Germany, and advantages for Southwestern Germany. Among women, the preventable cancer mortality has increased in Northwestern and Southwestern Germany after the German reunification but has decreased in Eastern Germany and converged to the pattern of Southwestern Germany. Similar patterns can be observed for females in amenable cancer mortality.

**Conclusions:** Although the “traditional” East-West gap in preventable cancer mortality was still evident in males, our study provides some hints for more regional diversity in avoidable cancer mortality in women. An establishing north-south divide in avoidable cancer mortality could alter the future trends in regional cancer-related mortality in Germany.

## Background

The regional variation in avoidable cancer mortality for Europe is known as a northwest (NW) to southeast (SE) gradient with higher mortality risk proportions for males (1–3). The NW to SE gradient in Europe is also accounted by the increasing smoking-related cancer incidence and the associated higher mortality risks especially for men in Southeast Europe. Otherwise, in North and West Europe, the cancer incidences tend to be stabilizing because of lower smoking prevalence in men and improved breast cancer screening technology in females (4).

On the regional level, Germany has experienced such geographic variations, with dramatic gaps in cancer-related mortality in the 1970s and 1980s. Prior to the German reunification, the cancer mortality was almost higher in East Germany, similar to the remaining mortality gap between Southeast and Northwest Europe (4, 5).

Within the last 25 years, East Germany has passed through an intensive economic and political alteration as well as the assimilation of the health care system under the same conditions of West Germany. This led to a partial adjustment of cancer related mortality in East Germany to the level of West Germany. Moreover, the regional pattern in avoidable cancer mortality known as a northeast-southwest divide still remains (6–8). Plausible explanations yield on a gender gap in avoidable cancer mortality, which tends to be higher in Eastern Germany. That is due to the remaining unfavorable life time risks in that region, especially in its rural areas.

In Eastern Germany, especially in its northern part, men potentially have higher risk of premature deaths because of less willingness of healthcare screening, high smoking and alcohol consumptions levels, overweight, and low physical activity (9–11). These harmful health behaviors also correspond with social factors and individual lifestyle choices in males.

Men in Eastern Germany are also more likely to be affected from poorer socioeconomic conditions, including redundancy, low income, and lower educational attainment that is also associated with unhealthy behavior (12, 13).

After German reunification, Eastern Germany experienced an increase of average life expectancy but also a decrease of birth rates and selective emigration of young and well-educated people, each resulting in an accelerated population aging. Regions with better job opportunities are attractive migration destinations. So far, migration is accounted as a selective health mechanism, with migrants usually being healthier than the stayers (14). In addition, the east-west migration balance is particularly negative among young women, resulting in a tremendous deficit in females among young to middle-aged adults in the rural areas of Eastern Germany (15).

The excess female emigration at county level can be explained with gender disparities in educational attainment that favored women (15). The female brain drain in Eastern Germany is also accompanied with less availability of potential partners for men (15, 16). The selective migration pattern accompanied by the loss in positive health risk factors as well as the discrepancy in sex-specific health behaviors are good indicators to explain regional variation in avoidable cancer mortality. Therefore, we use the gender gap in avoidable cancer mortality as a proxy for regional variation in the distribution of biological and non-biological factors (17, 18). Some studies argue that only 25% of the sex differences in mortality are attributable to biological differences (18). The major part can be explained by social and behavioral factors, which are in turn influenced by biological factors, however (19).

The major scope of our study was to analyze the regional long-term trends in avoidable cancer mortality for the period since the German reunification under consideration of a generalized approach for the avoidable cancer mortality definition that is suitable for the German regional perspective.

Moreover, we examined sex-specific disparities in avoidable cancer mortality to explain recent trends to explain the recent trends for three German super regions: Eastern, Northwestern, and Southwestern Germany.

## Data and Methods

Different approaches were used in literature to account for avoidable mortality that had involved different typologies of premature death. Generally, avoidable mortality can be referred to as a selection of causes of death that should be amenable to health care (amenable mortality) or as a selection of causes of death that should be avoidable through primary prevention (preventable mortality) (20). There are many different concepts of avoidable mortality but no commonly used classification of avoidable cancer mortality that includes both amenable and preventable conditions. Regarding the health service system and the national health risk profile in Germany, we have therefore, redistributed the avoidable cancer mortality classification to an approach that is more suitable for the case of Germany. This classification of avoidable cancer mortality and its division into preventable and amenable cancers basically follows the concepts of Nolte and McKee (21), Page et al. (22), Tobias et al. (23), Nolte and McKee (24), and Mackenbach et al. (25).

In the first group of preventable cancer mortality, we selected all cancer-related deaths that could be avoided or reduced through effective inter-sectoral health policies by means of primary prevention, especially with regard to smoking, unhealthy diet, and alcohol consumption. This involved cancers of lip, oral cavity, pharynx, esophagus, stomach, liver, larynx, lung, bronchus, trachea, and bladder.

The second group of amenable cancer mortality includes all cancer-related deaths that should be avoided or reduced through timely and effective health care regarding both, diagnosis and treatment. This involved cancers of colon and rectum, bone, skin, eye, and thyroid as well as Hodgkin's disease and leukemia and the sex-specific cancers of female breast, cervix, uterus, prostate, and testis (Table 1).

**Table 1:** Selection of causes of death.

	**Cause of death**	**Age**	**ICD-9**	**ICD-10**
Preventable cancer mortality	Cancer of lip, oral cavity, pharynx	0–74	140–149	C00–C14
	Cancer of esophagus	0–74	150	C15
	Cancer of stomach	0–74	151	C16
	Cancer of liver	0–74	155	C22
	Cancer of larynx^a^	0–74	161	C32
	Cancer of lung, bronchus and trachea	0–74	162	C33–C34
	Cancer of bladder^b^	0–74	188	C67
Amenable cancer mortality	Colorectal cancer	0–74	153–154	C18–C21
	Bone cancer^b^	0–74	170	C40–1
	Skin cancer	0–74	172, 173	C43, C44
	Eye cancer	0–74	190	C69
	Thyroid cancer	0–74	193	C73
	Hodgkin's disease	0–74	201	C81
	Leukemia^c^	0–44	204–208	C91–C95
→ Women only	Breast cancer	0–74	174	C50
	Cervical cancer	0–74	180	C53
	Uterine cancer	0–74	179, 182	C54, C55
→ Men only	Prostate cancer^a^	0–74	185	C61
	Testicular cancer	0–74	186	C62

*This classification is based on Page et al. (22), with the following exceptions*:

a *Laryngeal and prostate cancer were added according to Mackenbach et al. (25)*.

b *Bladder and bone cancer were added according to Tobias et al. (23)*.

c *Leukemia group was widened according to Nolte and McKee (21, 24)*.

For data analysis, we used the cause of death data for the period of 1990–2014 by sex, age, and regional level (16 German Bundesländer) as well as the corresponding year-end population numbers for the period of 1989–2014, provided by the Federal Statistical Office. In order to avoid fluctuations, three year values were used for all calculations. Thus, the analyses refer to the period from 1991 to 2013. The cause-specific death information on cancer was followed to the International Classification of Diseases (ICD) with ICD-9 codes up to 1997 and ICD-10 codes from 1998 onwards. We defined the following age- groups: 0–29, 30–39, 40–44, 45–49, 50–54, 55–59, 60–64, 65–69, and 70–74 years to estimate the age-specific death rates for specific cancers. The bounds of the first two age groups are broader due to the comparatively low numbers of cancer deaths at young ages. Older ages from 75 years upwards were not included “as “avoidability” of death and reliability of death certification become increasingly questionable at older ages” (21).

**Figure 1:**
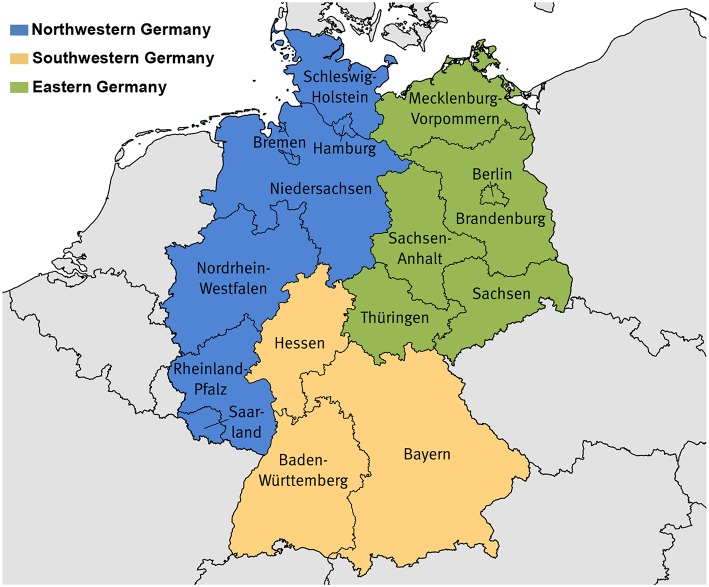
Composition of the study regions. Northwestern Germany: Bremen, Hamburg, Niedersachsen, Nordrhein-Westfalen, Rheinland-Pfalz, Saarland, Schleswig-Holstein; Southwestern Germany: Baden-Württemberg, Bayern, Hessen; Eastern Germany: Brandenburg, Berlin, Mecklenburg-Vorpommern, Sachsen, Sachsen-Anhalt, Thüringen. Base map: ©GeoBasis-DE/Bundesamt für Kartographie und Geodäsie.

For regional comparisons, we defined three geographic regions: Eastern, Northwestern, and Southwestern Germany (Figure 1). This geographical grouping is based on minimal internal group variation in the level of cancer mortality and its development over time as well as similarities in socioeconomic characteristics and age structure. Eastern Germany corresponds to the territory of the former GDR (East Germany), however including all of Berlin. Western Germany, thus excluding the city of Berlin, was divided into a northern and southern part to take account of systematic differences in mortality and socioeconomic characteristics, which otherwise would be overlaid. Doing this, both gradients, between north and south as well as between east and west, can be addressed.

The rates of preventable and amenable cancer mortality for men and women were computed for the period from 1991 to 2013 to observe sex-specific differences in specific cancers after the German reunification. We used standardized death rates to show all-cause and cause-specific mortality differences over time and between regions and sex. The main advantage of standardization is that death rates are no longer distorted by differences or changes in the age structure. Furthermore, standardized death rates are additive: “the sum of death rates by cause equals the death rate from all causes” (Meslé, 2006, p. 36) (26). Death rates with a directly standardized age and sex structure were therefore, computed for each region, differentiated by sex and cause of death group, based on textbooks like Preston et al. (27) but were adjusted for the use of 3 year periods and year-end population statistics:

(1) $$\begin{array}{l} SDR_{t}=\underset{x}{\sum }\left(\frac{D_{x;t-1}+D_{x;t}+D_{x;t+1}}{0.5\cdot N_{x;t-2}+N_{x;t-1}+N_{x;t}+0.5\cdot N_{x;t+1}}\cdot C_{x}\right) \end{array}$$

with *SD*R_*t*_ being the standardized death rate at time *t* (in years); D_*x*_ being the number of deaths at age *x*, N_*x*_ being the age-specific year-end population size, and C_*x*_ being the age-specific standard population. We chose the German 2011 Census as standard population without disaggregation by sex. As a statistical test for the standardized death rates, we calculated 95% confidence intervals according to Chiang (28), with the age-specific probability of death computed according to Farr (29). We interpreted mortality differences at time *t* as statistically significant when the confidence intervals of two regions did not overlap.

For relative comparison we used rate ratios from standardized death rate estimates, with Southwestern Germany as the reference population.

## Results

**Figure A1:**
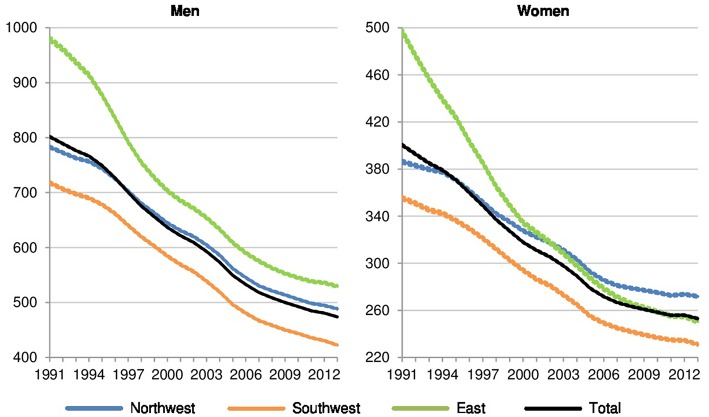
All-cause premature mortality per 100,000 in Northwestern, Southwestern, and Eastern Germany (standardized death rate, ages 0–74, years 1991–2013, 3 year values with 95% confidence intervals).

Overall, we find declining trends in preventable and amenable cancer mortality, comparable to the decrease in all-cause premature mortality (see Figure A1). In addition, avoidable cancer deaths contribute to one third of all premature deaths among men and to almost half of all premature deaths among women (see Table A1). Thus, we identify the decrease in avoidable cancer mortality as one of the major drivers for the decrease in German overall mortality at ages 0 to 74. The avoidable cancer deaths in all-cause premature mortality in Germany increased from 30 to 34% among men and rose from 41 to 48% among women between 1991 and 2013 due to the stronger decrease in other premature deaths. This relative growth is evident for all regions, but particularly for Eastern Germany, which shows an increase from 25 to 33% among men and from 34 to 45% among women. While avoidable cancer mortality is dominated by preventable cancer deaths among males, amenable cancer deaths are more prominent among females.

For men, preventable cancer mortality decreased by 29% in Eastern Germany, 34% in Northwestern Germany, and about 36% in Southwestern Germany between 1991 and 2013 (see Table A2). The relative decrease in amenable cancer mortality is similar among men: 28% in Eastern Germany, 32% in Northwestern Germany, and 36% in Southwestern Germany. Among women, however, the trend in preventable cancer mortality is diametrically opposed as there is an overall increase in all three regions. Amounting to 27%, the relative growth in preventable mortality is more present in Northwestern Germany, compared to 19% in Southwestern Germany and 1.4% in Eastern Germany. With regard to amenable cancer mortality, the pattern for women is similar compared to men, but showing a slightly higher relative decline: 40% in Eastern Germany, 35% in Northwestern Germany, and 37% in Southwestern Germany.

**Figure 2:**
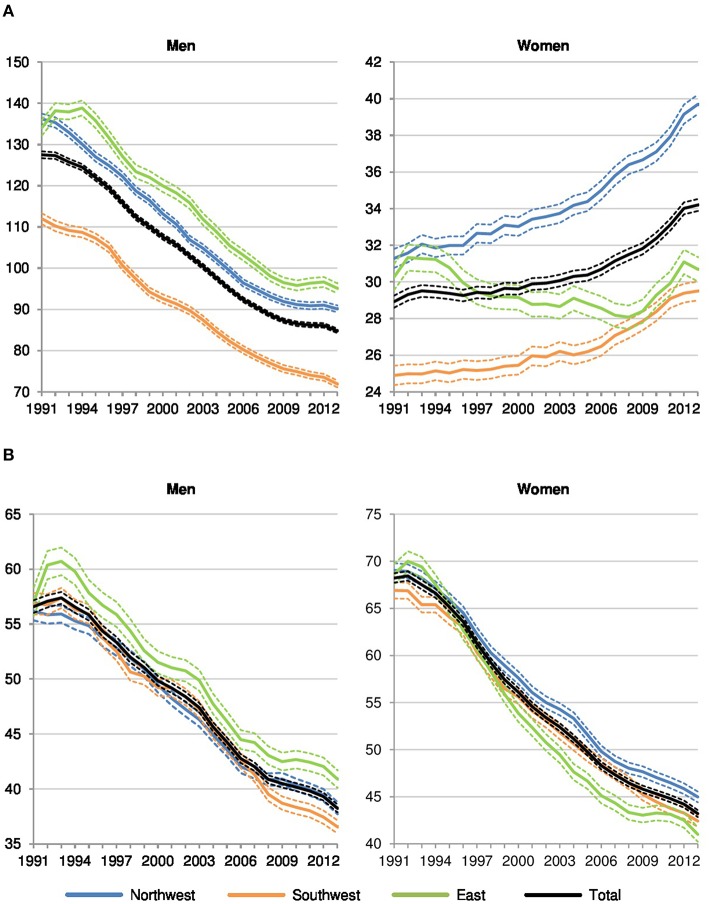
Preventable and amenable cancer deaths per 100,000 in Northwestern, Southwestern, and Eastern Germany (standardized death rate, ages 0–74, years 1991–2013, 3 year values with 95% confidence intervals). **(A)** Preventable cancer mortality. **(B)** Amenable cancer mortality.

Furthermore, we find remarkable sex-specific differences in *preventable cancer mortality* (Figure 2A) at the regional level. For men, in spite of a strong decrease in all regions, there is still a considerable east-west divide in the northern half of Germany but there is also an even more significant north-south divide in Western Germany. In 2013, preventable cancer mortality was significantly higher in Eastern (+32%) and Northwestern Germany (+25%) than in Southwestern Germany among men, approved by mortality rate ratios calculated on the basis of the standardized death rates (see Table A3). Among women, however, preventable cancer mortality has increased in all of Western Germany since reunification but had decreased in Eastern Germany until the late 2000s and since then it has almost converged to the pattern of Southwestern Germany. In comparison to Southwestern Germany, preventable cancer mortality was significantly higher for women in Northwestern (+34%) and Eastern Germany (+4%) at the end of the observation period.

Regarding *amenable cancer mortality* (Figure 2B), regional disparities for men and women are considerably smaller. The known east-west divide still dominates among men (+12% in Eastern Germany and +5% in Western Germany compared to the southwest in 2013) but a north-south gradient in Western Germany has developed in recent years as well. Among women, the pattern has become increasingly determined by a north-south gradient in Western Germany. In comparison to the southwest, amenable cancer mortality was significantly higher in the northwest (+6%), while Eastern Germany (−3%) showed the lowest level among the three regions in the last year of observation.

**Figure 3:**
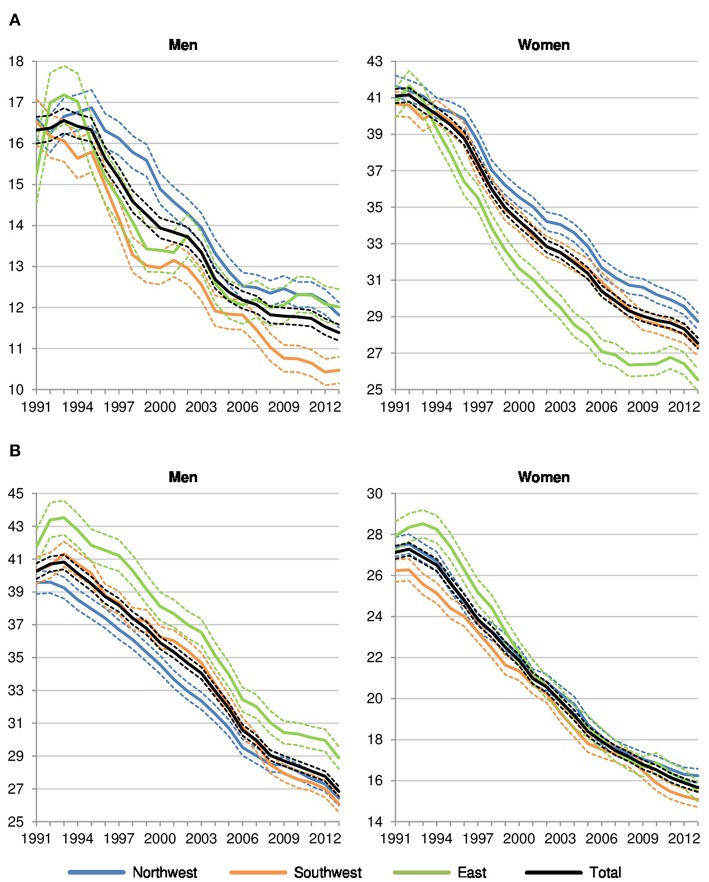
Sex-specific and other amenable cancer deaths per 100,000 in Northwestern, Southwestern, and Eastern Germany (standardized death rate, ages 0–74, years 1991–2013, 3 year values with 95% confidence intervals). **(A)** Sex-specific amenable cancer mortality. **(B)** Other amenable cancer mortality.

Dividing amenable cancer mortality into sex-specific and other cancer deaths, the level in *sex-specific amenable cancer mortality* is generally lower in men than in women since men-specific cancers are less frequent and less likely fatal than women-specific cancers (Figure 3A). Among men, there is a significant north-south divide to the advantage of the southwest but no systematic differences between Northwestern (+13% in 2013 compared to the southwest) and Eastern Germany (+15%). Among women, however, the pattern is completely different: Northwestern Germany (+5%) showed the highest rates, while Eastern Germany (−7%) showed the lowest values in comparison to the southwest.

Referring to *other amenable cancer deaths* (Figure 3B), the regional pattern which favors men and women in Southwestern Germany and disadvantages men in Eastern Germany and females in Northwestern Germany becomes visible again.

All in all, the analyses show that the “traditional” east-west gap in avoidable cancer mortality is still evident among men, while the north-south gradient has gained importance, with tendency to higher regional diversity. Among women, the northwest of Germany seems to be increasingly left behind the other two regions.

## Discussion

### Preventable Cancer Mortality

Down warding drifts in smoking prevalence for sex and between geographical regions are used to explain the lung cancer mortality disparities in Germany which are mostly responsible for the decline in preventable cancer mortality (30–32). Hence, some regional disparities in preventable cancer mortality for men and women remain evident. In contrast to the “traditional” east-west gradient (33, 34) in preventable cancer mortality, some regions in the East, especially for women, have not exceeded these regional mortality disadvantages as we know from males for the last decade.

With respect to the recent regional cluster affiliations, the female preventable cancer mortality, e.g., for the state of Sachsen, are more likely to take over the regional pattern of Southwestern Germany with more similarities to States of Bayern and Baden-Württemberg and Hessen (all three in vanguard position in preventable mortality) than to other regions in Northwestern Germany (including Bremen, Hamburg, Niedersachsen, Nordrhein-Westfalen, Schleswig-Holstein, Rheinland-Pfalz, Saarland). The assimilation process in Eastern Germany to the Southwestern German pattern can be explained with the loss of demographic impact of those age groups with high smoking epidemic in Eastern German women. Female Cohorts born between 1946 and 1950 have not showed any significant differences in smoking behavior in East Germany regarding their educational status (35). But women in West Germany predominantly have a higher smoking risk at the same time.

A recent study on the future perspective in smoking-related mortality in Germany (36) argues that the east-west convergence and especially the female mortality advantage in Eastern Germany in lung cancer-related mortality is caused by women born in the 1940s and 1950s who showed a comparatively low smoking prevalence. Possibly, the contemporary mortality advantage for women in Eastern Germany will change to the opposite as younger cohorts of Eastern German women show much higher smoking rates in comparison to both, previous cohorts and the West (36, 37).

The female lung cancer epidemic affecting the younger cohorts can be approximated. For women, a greater excess mortality for lung cancer is more plausible because of the increasing dominance of higher lung cancer rates in young and middle-aged women (36–38).

The remaining gender gap in preventable cancer mortality is due to lower risks of premature mortality in women. Moreover, the majority of women's sex-specific are amenable cancer deaths is dominated by breast cancer. For men, more unfavorable life-style risk factors including social and health behavior need to be examined to analyze male excess mortality (39, 40). Those risk factors are largely avoidable and contribute to the human, social, and economic burden that is largely invisible in the epidemiologic literature.

### Amenable Cancer Mortality

Amenable cancer mortality in Germany is also directed by colon/bowel cancer in men and breast cancer in women. The regional pattern in amenable deaths from cancer is more likely driven by the north-south gradient than by the east-west gap. Overall, the most favorable trends in amenable cancer mortality are still driven by improved therapeutic procedures and advancements in diagnosis and screening that positively affects typically more female cancers. A study from Brenner et al. (41) supports the general improvement in bowel cancer for men and women. After the introduction of screening colonoscopy starting at age 55 years in Germany, the incidence and mortality of bowel cancer have declined in the first decade of the twenty-first century (41). Although the mortality in bowel cancer has systematically declined for both sexes, females exhibit a survival benefit in this type of cancer compared to males. This can be explained the higher willingness of participation in cancer screening programs of women and their appropriated compliance during cancer therapy (41–43).

The most positive trends in amenable cancer mortality are revealed in breast cancer in Germany, because of tremendous improvement in treatment strategies of these cancer sites that had similar benefits for women in East and West Germany (44–47). Although breast cancer is defined as amenable cancer, there is a remaining gap between East and West Germany with unexpected lower incidences and mortality in breast cancer for the East. Those are not typically results only from improvements in therapeutic effects. Thereof, these regional disparities are explained with the variability in reproductive histories for East and West German women (48, 49). East German women born in the 1950s and 1960s were less frequently childless and their mean age at first childbirth was 5 years below the value for West German women (50). These two characteristics are important preventive factors in connection with breast cancer (49).

In addition, Eastern Germany, especially the federal state of Sachsen, took over a vanguard position in establishing regional (centralized) cancer registries including certified tumor centers/boards, which should positively affect the amenable cancer mortality trends. Consequently, there is a clear regional pattern in breast cancer mortality with the lowest mortality rates being found in Eastern Germany (Berlin, Brandenburg, Mecklenburg-Vorpommern, Sachsen, Sachsen-Anhalt, Thüringen) and the highest mortality rates in Northwestern Germany (including Schleswig-Holstein, Bremen, Hamburg, Niedersachsen) (51–53). Among women, there is a significant divide between Northwestern Germany and the other two study regions that is probably about to even increase in the near future. Thus, there is an evidence of a manifested Northwest-Southeast divide in amenable and preventable cancer mortality among women.

### Limitations

This study has applied a specific approach to capture the unique difficulties in defining avoidable cancer mortality for the regional comparison in Germany. At the same time, we recognized that some limitations remain. First, we could not consider an additional set of variables, especially all risk factors that were relevant in the development of avoidable cancer mortality. Consequently, this important information on the determinants of cancer mortality such as lifestyle (nutrition, physical activity, alcohol consumption) or environmental conditions (air pollution) was either not available for this recent statistical analyses or was not considered to be representative on the level of the three German super-regions. Socio-economic factors, which significantly influence the relevant risk factors and show considerable regional variations in Germany (40), are not included in official German data. Second, we did not use the data of the German cancer registries, which are recorded by the Association of Population-Based Cancer Registries (GEKID) and the Robert Koch Institute (RKI) every two years (54). Actually, these data offer a more precisely overview on cancer-related mortality and morbidity, such as the prevalence of 27 selected cancer sites, but they are only available for the period of 2004–2014 and not for all German Länder and would therefore not match our analytical and data setting.

Third, the long-term comparisons in cancer mortality are affected by time lags. This involves new treatment/therapeutic effects for amenable cancer mortality, developments in health policy, and changes of health risk behaviors for preventable cancer mortality, e.g., strict anti-smoking policy or healthy food programs (55). Amenable cancer mortality is also a quality benchmark for health care services. As an example, the advancement in treatment like adjuvant therapy or recent reimbursement policy in pharmaceuticals may contribute to the decline in cancer mortality. Fourth, we could not control for selective migration between the German regions. This phenomenon might be plausible to explain the healthy migrant effect and even the changes in exposure of health risks in some regions.

## Conclusions

Generally, our study showed that regional disparities in avoidable cancer mortality are more related to differences in risk-relevant behavior than differences in the effectiveness of health care. Regional differences in avoidable cancer mortality in Germany were much more significant among men, where the traditional east-west gradient was still evident. However, a general north-south divide was also evident and is especially significant in preventable cancer mortality among men. By contrast, a north-south gradient in Western Germany meanwhile was determined as the avoidable cancer mortality pattern among women particularly led to an alignment of Eastern Germany to the level of Southwestern Germany. Whereas, the male pattern could be explained by higher rates of smoking and alcohol abuse in the East, the female pattern was largely attributed to the high smoking rates of younger, especially Northwestern German cohorts who increasingly reach the cancer-relevant age.

There were structural deficits in Northwestern and Eastern Germany compared to the Southwest regarding the effectiveness of health care that however didn't touch Eastern German women that much, presumably because they were less often childless and thus less affected by breast cancer.

The regional pattern in avoidable cancer mortality has become much more diverse, with decreasing importance of the east-west gap and growing significance of a north-south divide.

German health policies should promote better access to medical care in geographic areas with low population density, especially in Eastern Germany and strengthen the improvement of male-specific compliance in primary prevention. Another provision should be the reduction of the male educational disadvantage which consequently affects the health-related lifestyle. Moreover, substantial investments in the labor market strategies to reduce the selective migration of highly educated young people from the east and north to the southern German metropolitan areas have to be considered.

## Data Availability

The datasets for this manuscript are not publicly available because Data must be requested by data transfer center. Requests to access the datasets should be directed to ronny.westerman@bib.bund.de.

## Author Contributions

RW and MM are responsible for the concept and design, drafting the article and revising it, and the final approval of the version to be published. MM performed all statistical analyses.

## Conflict of Interest Statement

The authors declare that the research was conducted in the absence of any commercial or financial relationships that could be construed as a potential conflict of interest.

## Appendix

**Table A1:** Share of preventable and amenable cancer deaths in all-cause mortality at ages 0–74 in 1991 and 2013 for men and women in Northwestern, Southwestern, Eastern, and total Germany (in %).

	**Preventable cancer mortality**	**Amenable cancer mortality**			**Total avoidable cancer mortality**
		**Total**	**Sex-specific**	**Other**	
Men 1991				
Southwest	15,6	7,9	2,3	5,6	31,4
Northwest	17,4	7,2	2,1	5,1	31,8
East	13,7	5,8	1,6	4,3	25,3
Total Germany	15,9	7,1	2,0	5,0	30,0
Men 2013
Southwest	17,0	8,7	2,5	6,2	34,3
Northwest	18,4	7,8	2,4	5,4	34,1
East	18,0	7,7	2,3	5,5	33,4
Total Germany	17,9	8,1	2,4	5,7	34,0
Women 1991
Southwest	8,1	17,9	10,8	7,1	43,8
Northwest	7,0	18,8	11,4	7,4	44,7
East	6,1	13,8	8,2	5,6	33,7
Total Germany	7,2	17,0	10,3	6,8	41,3
Women 2013
Southwest	12,8	18,4	11,8	6,5	49,5
Northwest	14,6	16,5	10,6	6,0	47,7
East	12,3	16,4	10,2	6,2	45,0
Total Germany	13,5	17,1	10,9	6,2	47,7

**Table A2:** Relative trend in preventable and amenable cancer mortality and all-cause mortality at ages 0–74 between 1991 and 2013 for men and women in Northwestern, Southwestern, Eastern, and total Germany (in %).

	**Preventable cancer mortality**	**Amenable cancer mortality**			**Premature all cause mortality**
		**Total**	**Sex-specific**	**Other**	
Men					
Southwest	–35,8	–35,7	–36,6	–35,3	–41,2
Northwest	–33,8	–31,9	–28,8	–33,2	–37,6
East	–29,1	–28,2	–21,2	–30,8	–46,0
Total Germany	–33,5	–32,4	–30,2	–33,3	–40,9
Women
Northwest	+26,9	–34,8	–31,0	–40,7	–29,6
Southwest	+18,5	–36,6	–32,8	–42,5	–35,0
East	+1,4	–40,3	–37,3	–44,6	–49,6
Total Germany	+18,2	–36,7	–33,0	–42,2	–36,8

**Table A3:** Ratio of the standardized death rates in preventable and amenable cancer mortality and all- cause mortality at ages 0–74 in 1991 and 2013 for men and women in Northwestern, Southwestern, Eastern, and total Germany.

	**Preventable cancer mortality**	**Amenable cancer mortality**	**Premature all cause mortality**
		**Total**	**Sex-specific**	**Other**	
Men 1991
Southwest	1	1	1	1	1
Northwest	1,22	0,99	1,00	0,98	1,09
East	1,20	1,00	0,92	1,04	1,36
Total Germany	1,14	1,00	0,99	1,00	1,12
Men 2013
Southwest	1	1	1	1	1
Northwest	1,25	1,05	1,13	1,01	1,16
East	1,32	1,12	1,15	1,11	1,25
Total Germany	1,18	1,05	1,09	1,03	1,12
Women 1991
Southwest	1	1	1	1	1
Northwest	1,26	1,03	1,02	1,04	1,09
East	1,22	1,03	1,00	1,06	1,40
Total Germany	1,16	1,02	1,01	1,03	1,13
Women 2013
Southwest	1	1	1	1	1
Northwest	1,35	1,06	1,05	1,08	1,18
East	1,04	0,97	0,93	1,03	1,08
					
